# Development of the informed health choices resources in four countries to teach primary school children to assess claims about treatment effects: a qualitative study employing a user-centred approach

**DOI:** 10.1186/s40814-020-00565-6

**Published:** 2020-02-10

**Authors:** Allen Nsangi, Daniel Semakula, Sarah E. Rosenbaum, Andrew David Oxman, Matt Oxman, Angela Morelli, Astrid Austvoll-Dahlgren, Margaret Kaseje, Michael Mugisha, Anne-Marie Uwitonze, Claire Glenton, Simon Lewin, Atle Fretheim, Nelson Kaulukusi Sewankambo

**Affiliations:** 1 College of Health Sciences, Makerere University, Kampala, Uganda; 2 University of Oslo, Oslo, Norway; 3 Centre for Informed Health Choices, Norwegian Institute of Public Health, Postboks 222 Skøyen, 0213 Oslo, Norway; 4 Infodesignlab, Oslo, Norway; 5 Great Lakes University of Kisumu, Kisumu, Kenya; 6 University of Rwanda, Kigali, Rwanda; 7 Health Systems Research Unit, South African Medical Research Council, Cape Town, South Africa

**Keywords:** User-centred design, User-testing, User experience, Pilot study, Critical thinking, Critical appraisal, Teaching, Education

## Abstract

**Background:**

People of all ages are flooded with health claims about treatment effects (benefits and harms of treatments). Many of these are not reliable, and many people lack skills to assess their reliability. Primary school is the ideal time to begin to teach these skills, to lay a foundation for continued learning and enable children to make well-informed health choices, as they grow older. However, these skills are rarely being taught and yet there are no rigorously developed and evaluated resources for teaching these skills.

**Objectives:**

To develop the Informed Health Choices (IHC) resources (for learning and teaching people to assess claims about the effects of treatments) for primary school children and teachers.

**Methods:**

We prototyped, piloted, and user-tested resources in four settings that included Uganda, Kenya, Rwanda, and Norway. We employed a user-centred approach to designing IHC resources which entailed multiple iterative cycles of development (determining content scope, generating ideas, prototyping, testing, analysing and refining) based on continuous close collaboration with teachers and children.

**Results:**

We identified 24 Key Concepts that are important for children to learn. We developed a comic book and a separate exercise book to introduce and explain the Key Concepts to the children, combining lessons with exercises and classroom activities. We developed a teachers’ guide to supplement the resources for children.

**Conclusion:**

By employing a user-centred approach to designing resources to teach primary children to think critically about treatment claims and choices, we developed learning resources that end users experienced as useful, easy to use and well-suited to use in diverse classroom settings.

## Article summary

**Table:** 

**Strengths and limitations of this study** **Strengths** • We used a user-centered design approach with a multi-disciplinary team. • We engaged end-users in the entire development process from brainstorming to piloting. • Non stringent grant conditions permitted ample time to generate and prototype ideas and then iteratively design the resources. **Limitations** • Time constraints in trying to synchronise the design schedule with the already busy school schedule	

## Summary box

**Table:** 

What is already known:	
• There is an information overload regarding unsubstantiated claims of benefits and harms of treatments • People generally lack the skills to assess the reliability of treatment claims • Lack of resources to teach critical thinking particulary appraising treatment claims in primary schools in both low and high-income countries.	
What are the new findings:	
• Use of a user-centered design approach to design resources • Benefits of multi-stake holder collaboration in the design process	
How might it impact on clinical practice in the foreseeable future?	
• We designed useful, understandable and transferable resources to teach critical thinking that children and teachers found relevant and easy to use in their particular contexts.	

## Background

People of all ages, in low- and high-income countries, are flooded with both reliable and unreliable information about how to care for their health, including claims about the benefits and harms of treatments (any action intended to improve health) [1]. Unreliable claims come from many sources, including experts, advertisements and family [2]. People’s beliefs in unproven claims about treatments can lead to harm and waste [2]. Although this problem is global, people with fewer resources to spend on unnecessary treatments are disproportionately affected.

Many studies have found that people’s ability to understand and assess health information is often lacking [1, 3–5], although there are limitations in how this has been measured [6]. The Informed Health Choices project aims to enable people to assess claims about the effects of treatments, beginning with primary school children.

### Why target primary school children?

Research has suggested that children between the ages of 10 and 12 are capable of learning critical appraisal skills [7], and teaching these basic skills is already part of the curricula in some countries [8]. It is possible to reach a large segment of the population before they drop out of school, as many do after primary level in low-income countries [9–11]. Finally, teaching children to assess information about treatment effects can lay a foundation for them to make informed health decisions when they grow older, as patients, future health professionals, policymakers and citizens.

A recent overview of six systematic reviews on education interventions in under resourced countries included 227 studies in total, but none of these studies addressed health or science literacy, or critical thinking more broadly [12]. Systematic reviews of teaching children critical appraisal skills in health also have not found studies of strategies for teaching these skills to primary school children in both low and high income settings [6, 13].

We developed the Informed Health Choices (IHC) primary school resources to help children begin to learn critical appraisal skills required to assess benefits and harms of treatments. Our objective was to design resources that children and teachers experienced as useful, easy to use, understandable, credible, desirable, and well-suited in classroom settings. In this article, we describe the development of these resources.

## Methods

Researchers in Norway, the United Kingdom, Uganda, Kenya, and Rwanda collaborated to develop and evaluate learning resources for school children and their parents in 2013 to 2017. This included development of a podcast for parents [14]; development of the CLAIM Evaluation Tool for measuring people’s ability to assess treatment claims [15]; a randomised trial of the effects of using the (IHC) primary school resources [16]; a randomised trial of the effects of listening to the podcast [17]; and a process evaluation [18].

### Participants and setting

While most of the piloting, user testing, and feedback took place in Uganda, we wanted to create resources that could also be used in other countries. Therefore, we also carried out piloting and user-testing of Version 2 of the resources in two East African countries (Rwanda and Kenya) and in one high-income country (Norway).

For pilot testing, we recruited schools that were geographically accessible to our team, taught in English, and were willing to make time. We contacted head teachers, who identified science teachers and classes of children who were prepared to pilot the resources. To recruit user-test participants, we used purposeful sampling to include year 5 students (10 to 12-year-olds) and their teachers. Table 1 describes the participants, and (Additional file 1) describes which participants we included in each step of the development work.

**Table 1:** Participants

Participants	Description
Researchers, teachers and journalists from several countries	The initial brainstorming session at the kick-off meeting for the project included 18 people from Indonesia, Nepal, Norway, Uganda, and the United Kingdom with various backgrounds, including teachers, journalists, medical doctors, information designers, anthropologists, public health specialists, and health service researchers.
A national advisory board in Uganda	The advisory board for the project included fifteen members (2 women and 13 men) representing various stakeholders, including the Ministry of Education, Ministry of Health, and Ministry of Gender, Labour and Social Development (which is responsible for children’s affairs in Uganda), and representatives from civil society and local government.
A teachers’ network in Uganda	The teachers’ network included 24 Ugandan primary school teachers (10 women and 14 men) in active practice from both rural and urban schools that were either government or privately owned [19].
Schools in Uganda	Of the five schools that participated in both phases of the development process (pilot and user-testing), four were government (public) schools and one was a private school. One of the government schools was one of the biggest schools in the country, with a teacher-student ratio of 1:250. The other three government schools were of typical size, with a teacher-student ratio of 1:120. The private school was small, with a teacher-student ratio of 1:35, in comparison to the average Uganda school with a teacher student ration of 1:70. For logistic purposes (travel by the investigators), three of the schools that participated were located in the Kampala urban area and two were in the semi-urban area surrounding Kampala. All of the schools were poorly equipped. Lessons were in English, although English was not the primary language spoken at home for most of the children. All of the classes were year-5, for which the official starting age is 10.
A school in Kenya	The school in Kenya was a government school with about 400 children attending year-1 to year-8 classes. The year-5 children were mostly between 10 and 14 years old.
A school in Rwanda	The school in Rwanda was a government (public) primary and secondary school with over 3000 children. The language of instruction was English and the age range for year-5 children was 10 to 15 years old.
Children in Norway	A convenience sample of four 12-year-old girls who knew each other, from a nearby school participated in piloting a series of eight games together with the research team, partly in Norwegian and partly in English.
A school in Norway	The school in Norway was a private international school, with 18 children in each class. It was well equipped. Lessons were in English, although English was not the primary language spoken at home for most of the children. The three classes were year-7, for which the typical starting age is 11.

### Developing the resources

**Fig. 1:**
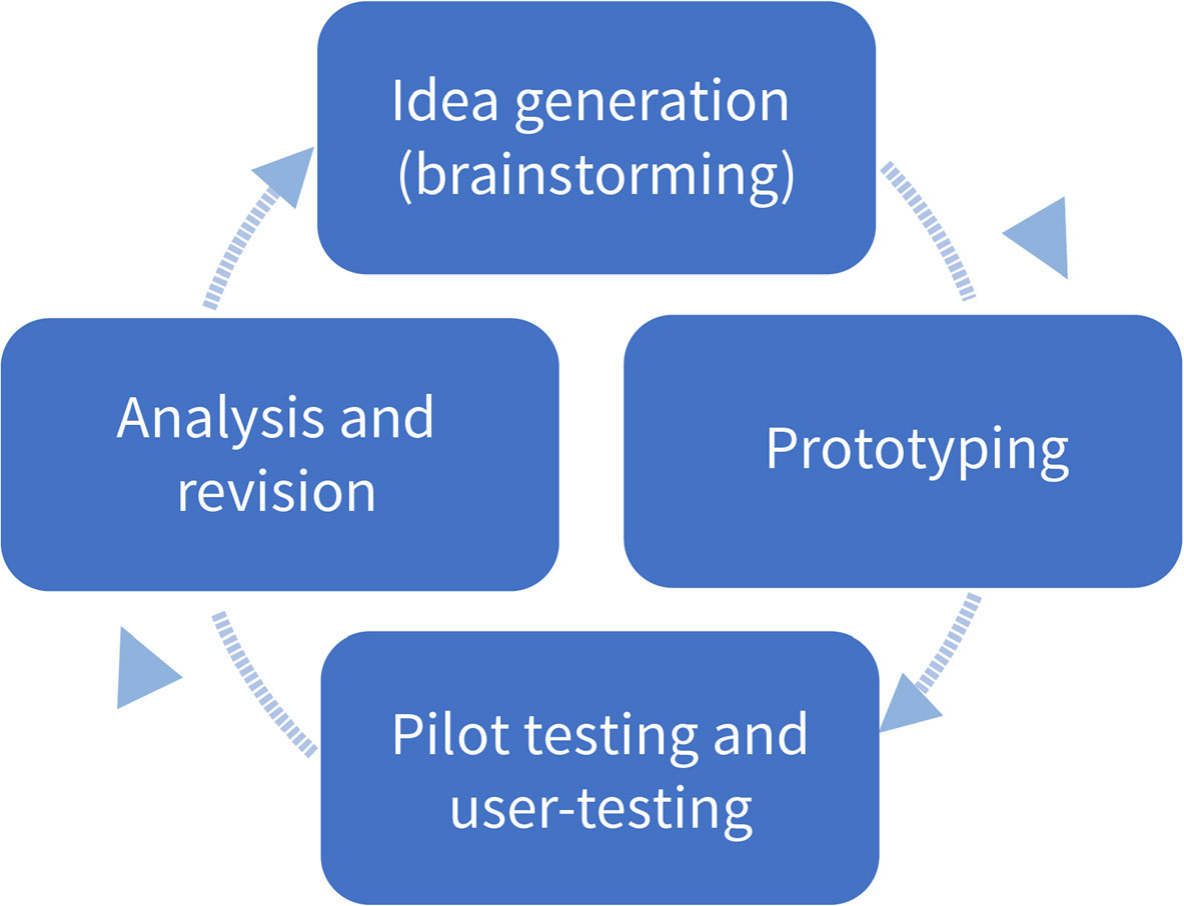
User-centred design development in multiple iterative cycles

We employed a user-centred approach to designing the IHC primary school resources [19–22]. User-centred design is characterised by multiple iterative cycles of development (Fig. 1).

Our starting point for developing these learning resources was to create a list of 32 Key Concepts that people need to understand and be able to apply to assess claims about treatment effects and make informed health choices [15]. A network of teachers in Uganda assessed the relevance of these concepts for primary school children during the prioritisation process and determined that 24 of these concepts were relevant to primary school children in Uganda [23].

#### Idea generation and prototyping

We used “creative thinking” in the idea generation and prototyping process. Creative thinking focuses on exploring ideas, generating possibilities and looking for many options [24]. This contrasts with critical assessment, which focuses on analysis, figuring out the answer and eliminating incorrect options. Both types of thinking were necessary for generating appropriate options for the resources we developed.

We needed to bring stakeholders and end users as close as possible into all phases of the work. This was particularly important since none of us belonged to the end user groups we were developing resources for (primary school children and their teachers). We included teachers as close collaborators through brainstorming [25] and prototyping workshops, and sought early feedback from children through workshops and school visits. We conducted multiple workshops in Uganda and Norway with the IHC research team and a network of teachers in Uganda [23]. These workshops resulted in ideas and insights about the context and stakeholders, sketches, and prototypes. We selected and developed ideas that we thought had the most potential to create new prototypes. These prototypes formed the basis for the next phases of pilot testing and user-testing.

#### Pilot testing and user-testing

We pilot tested early prototypes in workshops with teachers and children and through school visits in Uganda and Norway, using participatory observation to facilitate participants’ engagement. We piloted later, more complete prototypes (Version 1 in Uganda, and Version 2 in Uganda, Rwanda, Kenya and Norway) using non-participatory observations of the classroom lessons to explore how teachers and children used these resources. We used a structured form to record observations (Additional file 2), as well as video and still photography.

We also carried out user-test interviews with individual children and teachers to explore their experience when interacting with our resources [19]. User-testing originated from human computer interaction, where effectiveness and efficiency of a product is measured in relation to personal satisfaction of the individual using the product. We used a qualitative approach, building on Rosenbaum’s adaptation of Peter Moville’s honeycomb framework of user experience [19–22, 25–27] to develop the interview guides. We focused on six facets of the users’ experiences: usefulness, ease of use, understandability, credibility, desirability, and identification (Table 2) [19].

**Table 2:** Six facets from the honeycomb framework

Facet	Description
Usefulness	Does this product have practical value for this user?
Usability	How easy and satisfying is this product to use?
Understandability	Does the user recognise what the product is and do they understand the content? (own subjective experience of understanding)
Credibility	Is it trustworthy?
Desirability	Is it something the user wants - has a positive emotional response to?
Identification	Does the user feel the product is for” someone like me” or is it alienating/foreign-feeling? (e.g. age, gender, culture–appropriate)

#### Analysis and revisions

We used a framework analysis approach to guide data collection and analysis. We entered observations from the pilot testing and feedback from the user-testing into a spreadsheet after each round of testing. Between two and five researchers from the IHC working group independently coded each observation based on the importance of the finding (Table 3) and its implications for changes to the learning resources.

**Table 3:** Coding of the importance of observations and feedback

Code	Description
Very important negative finding (“show stopper”)	A problem that we should address for the resources to be effective
Important negative finding	A problem that we should probably address for part of the resources to be effective
Negative finding	A problem that we can easily address and probably will not prevent the resources from being effective
Very important positive finding	Praise that probably should inspire changes
Important positive finding	Praise that maybe should inspire changes
Positive finding	Praise that probably should not inspire changes
Very important constructive finding	A suggestion that probably should inspire changes
Important constructive finding	A suggestion that maybe should inspire changes
Constructive finding	A suggestion that probably should not inspire changes

The coding was combined in a single spreadsheet, discussed, and a consensus was reached. Based on these findings, we generated a list of potential problems and suggestions for changes. We discussed major problems and brainstormed solutions to those problems with the rest of the IHC working group. After agreeing on the changes needed, we created new prototypes to be piloted and user-tested.

We did not collect or analyse any quantitative data.

**Fig. 2:**
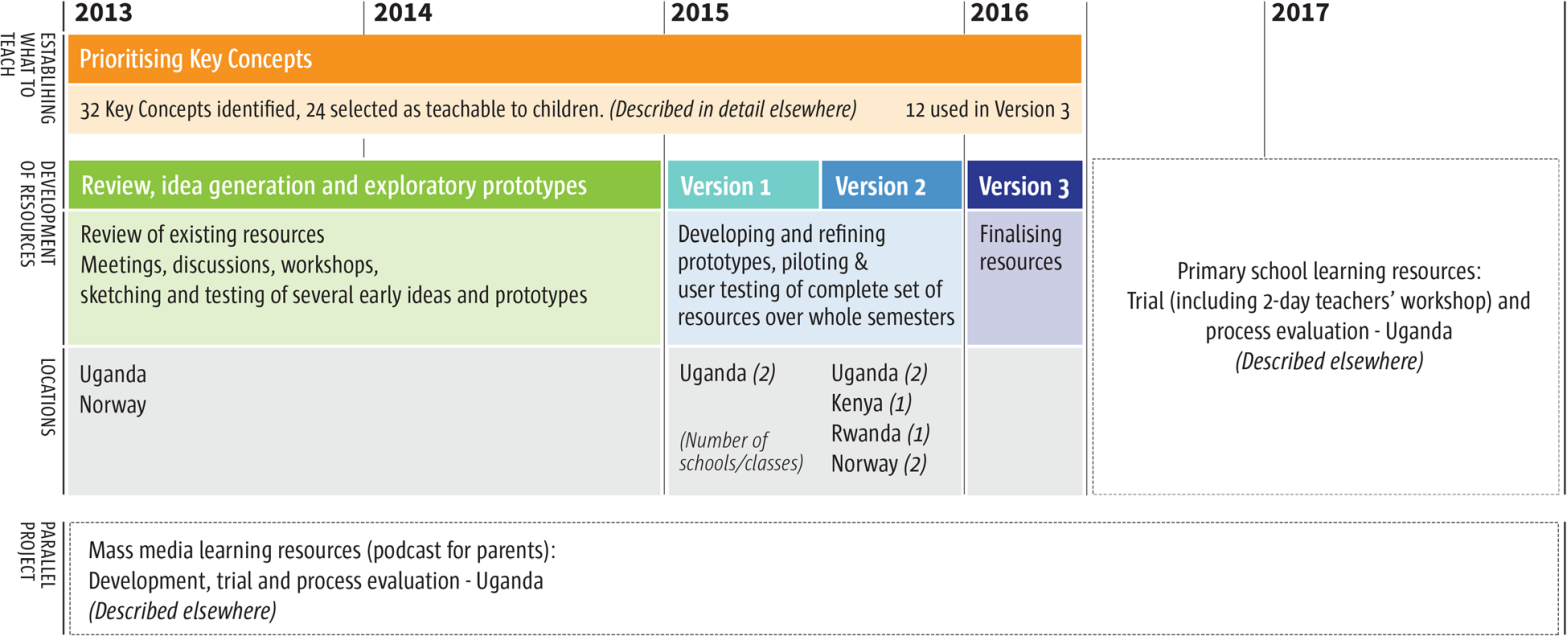
Development timeline

A timeline showing the development process, beginning with prioritisation of the Key Concepts is shown in (Fig. 2), and each step is summarised in (Additional file 1).

### Patient and public involvement statement

End users such as teachers on the network, policy makers on the advisory panels and primary school children participated in the development process by providing structured feedback of the resources at various iteration stages.

## Results

### Prioritising key concepts

We started with the list of 32 Key Concepts identified at the beginning of this project [15]. However, although 24 of these concepts were deemed relevant for primary school children, teaching all 24 concepts proved to be too much to learn in a school term. The early prototypes we created had too many concepts per lesson and took too long to teach in a normal school hour (40 min). We also observed that the teachers needed time to repeat material from previous lessons. We therefore reduced the number of concepts addressed in the final version of the resources to 12 (Table 4), as described in (Additional file 3). The other 12 concepts could be taught in a subsequent school term.

**Table 4:** Key Concepts that are relevant for primary school children

*Key Concepts taught in The Health Choices Book*	
CLAIMS: ARE THEY JUSTIFIED?	
• Treatments may be harmful	
• Personal experiences or anecdotes (stories) are an unreliable basis for assessing the effects of most treatments	
• Widely used treatments or treatments that have been used for a long time are not necessarily beneficial or safe	
• New, brand-named, or more expensive treatments may not be better than available alternatives	
• Opinions of experts or authorities do not alone provide a reliable basis for deciding on the benefits and harms of treatments	
• Conflicting interests may result in misleading claims about the effects of treatments	
COMPARISONS: ARE THEY FAIR AND RELIABLE?	
• Evaluating the effects of treatments requires appropriate comparisons	
• Apart from the treatments being compared, the comparison groups need to be similar (i.e. ‘like needs to be compared with like’)	
• If possible, people should not know which of the treatments being compared they are receiving	
• Small studies in which few outcome events occur are usually not informative and the results may be misleading	
• The results of single comparisons of treatments can be misleading	
CHOICES: MAKING INFORMED HEALTH CHOICES	
• Treatments usually have beneficial and harmful effects	
*Other Key Concepts prioritised for children*	
CLAIMS: ARE THEY JUSTIFIED?	
• An outcome may be associated with a treatment, but not caused by the treatment	
• Increasing the amount of a treatment does not necessarily increase the benefits of a treatment and may cause harm	
• Hope or fear can lead to unrealistic expectations about the effects of treatments	
• Beliefs about how treatments work are not reliable predictors of the actual effects of treatments	
• Large, dramatic effects of treatments are rare	
COMPARISONS: ARE THEY FAIR AND RELIABLE?	
• People in the groups being compared need to be cared for similarly (apart from the treatments being compared)	
•	
• It is important to measure outcomes in *everyone* who was included in the treatment comparison groups	
• Results for a selected group of people *within* fair comparisons can be misleading	
• Reviews of treatment comparisons that do not use systematic methods can be misleading	
• Well done systematic reviews often reveal a lack of relevant evidence, but they provide the best basis for making judgements about the certainty of the evidence	
CHOICES: MAKING INFORMED HEALTH CHOICES	
• Fair comparisons of treatments should measure outcomes that are important	

### Review, idea generation and exploratory prototypes

This phase lasted two years and was highly exploratory. In addition to the workshops and prototype development described below, we also engaged regularly with the teachers’ network and the Uganda National Advisory board.

#### Reviewing existing resources

We collected ideas from our own experiences teaching critical appraisal to children [28] and adults (including health professionals, policymakers, journalists, and patients), a systematic review of interactive resources for teaching critical appraisal skills to consumers [29], and searching the TES database and other sources such as google scholar for relevant resources.

We had a series of brainstorming sessions with members of the research team, informed by the resources that we found and workshops that we conducted with teachers and other researchers. In October 2015, we organised an international workshop with others interested in helping people to assess claims about treatments where a variety of resources was discussed. This workshop led to the development of the Critical thinking and Appraisal Resource Library (CARL) [30]. The Critical thinking and Appraisal Resource Library (CARL) is a platform to collect and distribute freely-available learning resources intended to help people think critically about treatment claims.

#### Idea generation workshop with researchers, teachers and journalists

In this meeting, we generated a broad range of ideas, from holding science fairs to creating interactive videos. Some ideas we generated were: use of drama and storytelling, board and field games, getting children to run a trial over several months, building a collection of familiar examples, translating already existing resources into local languages, holding teacher training workshops.

The main challenges we identified included: the need to teach the teachers; developing resources that would work in schools without digital equipment and where languages other than English were spoken; finding time in the curriculum, and gaining buy-in from stakeholders (including teachers, parents, and policymakers).

We decided to focus the next step on developing interactive classroom games that could be carried out with simple readily available equipment, like blackboards.

#### Pilot testing games in classrooms

We developed presentation materials and prototypes for two games to be used in classrooms: tossing coins to explain the concept of ‘chance’ and a game involving comparing the effects of two different coloured candies to explain Key Concepts related to fair comparisons. Children worked together in small groups. We piloted the games in classes at three schools – one in Norway and two in Uganda, with numbers of children ranging from 30 to 129. We participated by taking the role of teachers.

The children clearly enjoyed these activities. They were engaged, asked relevant questions and came up with some of the concepts by themselves, like blinding. But the exercise tended to get out of hand when the children were required to work independently and discuss in small groups. This was a problem even in Norway, despite the smaller class size. The children also needed more structured materials and more facilitation than we had anticipated. Their understanding of the concept ‘fair’ was different than what we meant when talking about fair comparisons, which we referred to initially as “fair tests”. One child said:

“For the test to be a fair test, everyone should get a candy”*.*

Despite being encouraged by the apparent ability of the children to understand many of the concepts, we also experienced first-hand that it could be challenging to explain the concepts correctly, even with semi-structured presentation materials. Teachers who were unfamiliar with the concepts would likely have even more difficulty.

#### Prototyping and pilot testing in Uganda and Norway

We conducted a prototyping workshop with 24 members of the teachers’ network in Uganda, piloted a game at a school in Uganda and an international school in Norway, and piloted a series of eight games with four 12 year-old girls in Norway (Additional file 4).

We found that although some of the games appeared to be promising, several were still too complicated to carry out in large classrooms. We also still had not solved the problem of how to transfer our presentation role to a teacher who was unfamiliar with the concepts without relying on electronic equipment like PowerPoint or video.

We decided to produce a highly-structured narrative for presenting the Key Concepts, which the teacher and children would read together, as well as a guide for the teacher. We decided to make a narrative in the form of a comic book with game-like activities and individual exercises included. We developed five characters to build the story around: two school children, two professors and a parrot who made unreliable claims about treatments, in an unspecified setting that would look like a rural east African village. Our thinking was that the narrative and use of drawings would engage the children, make the Key Concepts easier to understand, and help them to retain what they learned [31, 32].

Despite many problems, there was enough enthusiasm for the comic format that we felt it had the potential to work in a Ugandan classroom. But it needed to be much simpler, and the explanation of each concept needed more space. Based on our findings and observations, we agreed to make the following changes in the next version:

- Rewrite and redraw the children’s book with
  - A much simpler story, language, and drawings; and shorter chapters with larger text
  - No complicated comic language
  - Glossary explanations where terms first occur, with definitions translated to Luganda
  - Examples that were less likely to be misleading
  - Simplify all activities so they would not require extra resources, or require being outdoors
- Revise the teachers’ guide by
  - Making it more like a recipe
  - Integrating the children’s book in the teachers’ guide to facilitate the lesson flow

We decided to produce the final version of the books in colour, but continued sketching prototypes in black-and-white.

### The IHC primary school resources

**Fig. 3:**
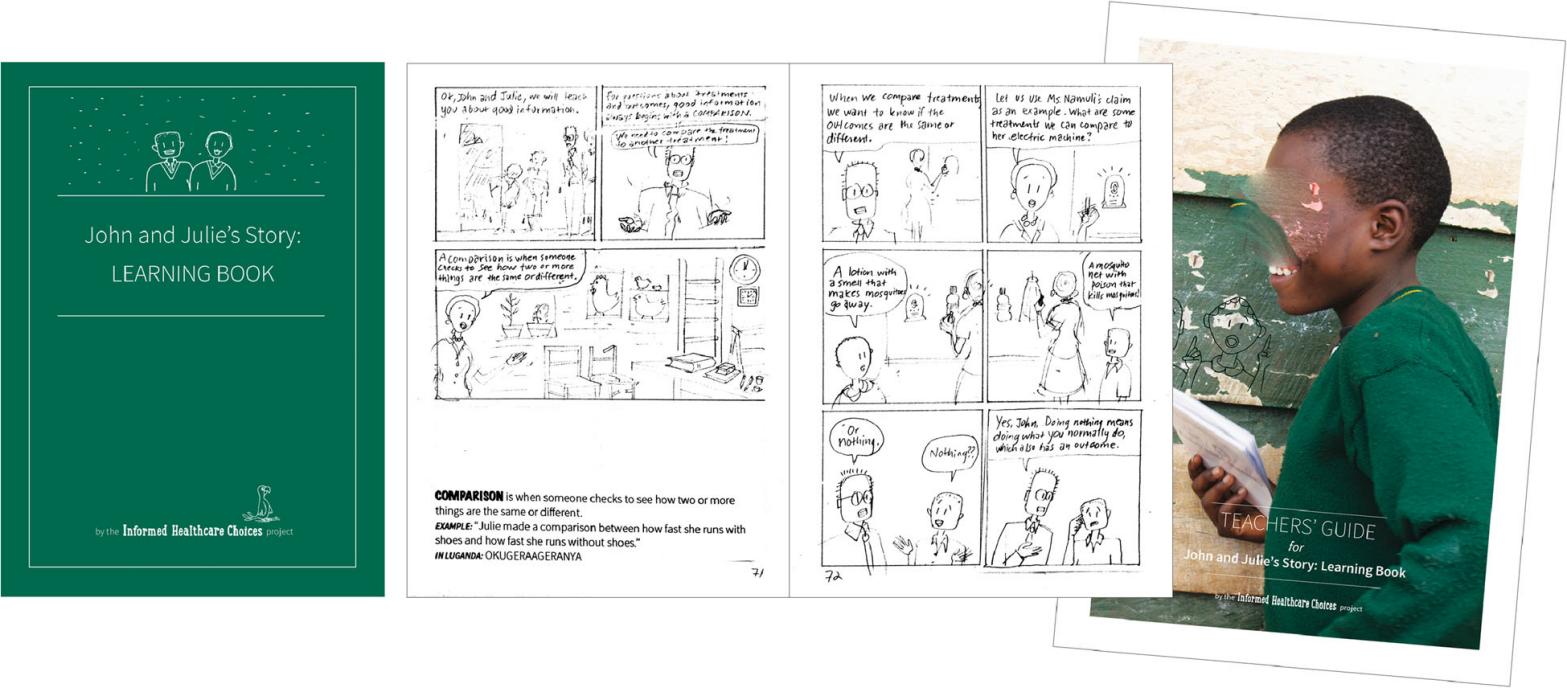
Version 1 prototype of the IHC primary school resources

We created three complete versions of the children’s book and teachers’ guide. The first version had 11 chapters (Fig. 3). We carried out pilot tests and user-testing at two schools in Uganda. Based on the users’ experiences (Additional file 5), we made the following changes to the next version of the children’s book:

- More emphasis on “critical thinking” rather than becoming a “junior researcher”
- Add a new first chapter that clarifies the purpose of the book, introduces some of the basic vocabulary in more depth (“health” “treatments”, “effects” and “claims”).
- Make usefulness more apparent by placing the story in the context of real life decision-making (e.g. the children in the book making a poor decision in the beginning and a more informed decision at the end)
- Adjust chapter content so that lessons could be fitted into 40-min periods
- Repeat learning goals from the previous chapter and introduce new characters at the beginning of each chapter
- Continue to simplify vocabulary; add a glossary in the back of the book
- Use a computer font instead of handwriting
- Add arrows to the comic cells to indicate reading direction
- More expressive and differentiated characters

We agreed on the following changes to the Teachers’ guide:

- Introduce more structure
- Add more background information, both about the purpose of the resources and about the key concepts covered in each chapter
- Decrease the number of lesson goals in each chapter

**Fig. 4:**
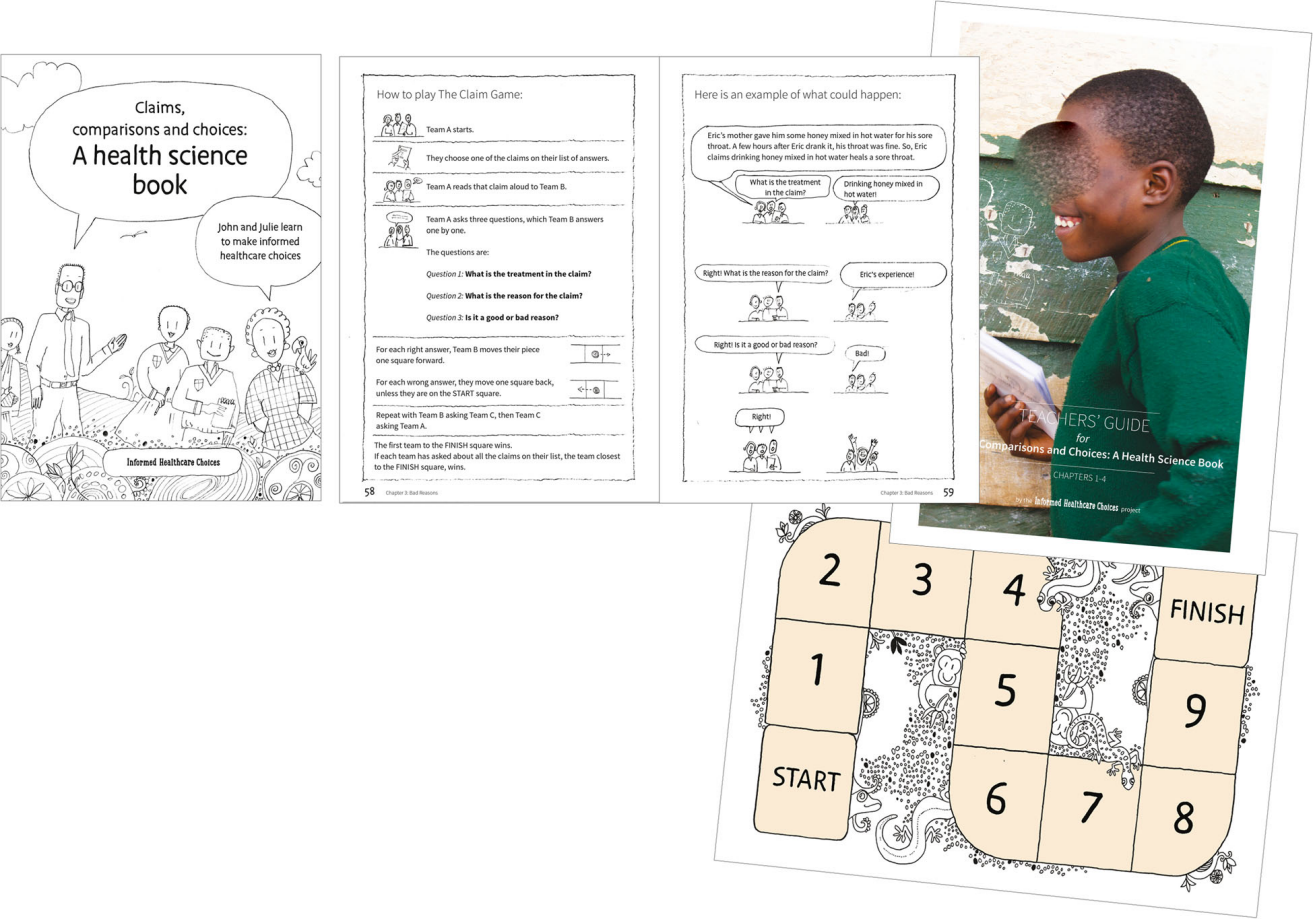
Version 2 prototype of the IHC primary school resources

We created Version 2 of the children’s book and teachers’ guide (Fig. 4), which had 10 chapters divided into two books. We carried out pilot tests and user-testing at schools in Uganda, Rwanda, Kenya, and Norway.

The most important problem that we identified was insufficient time to teach all the content included in Version 2. Based on the users’ experiences (Additional file 5), we agreed to make the following changes in the next version of the children’s book:

- Revise the CLAIM game and make it less demanding on the teacher to organise
- Introduce a glossary that explains all the new terms in the children’s book
- Reduce the number of exercises at the end of each lesson
- Further simplify or remove chapters that were difficult for the children to understand, like chapter 8 on “careful summaries” (systematic reviews)

We agreed to make the following changes in the teachers’ guide:

- Add more examples
- Revise and restructure the content and add a structured lesson plan

**Fig. 5:**
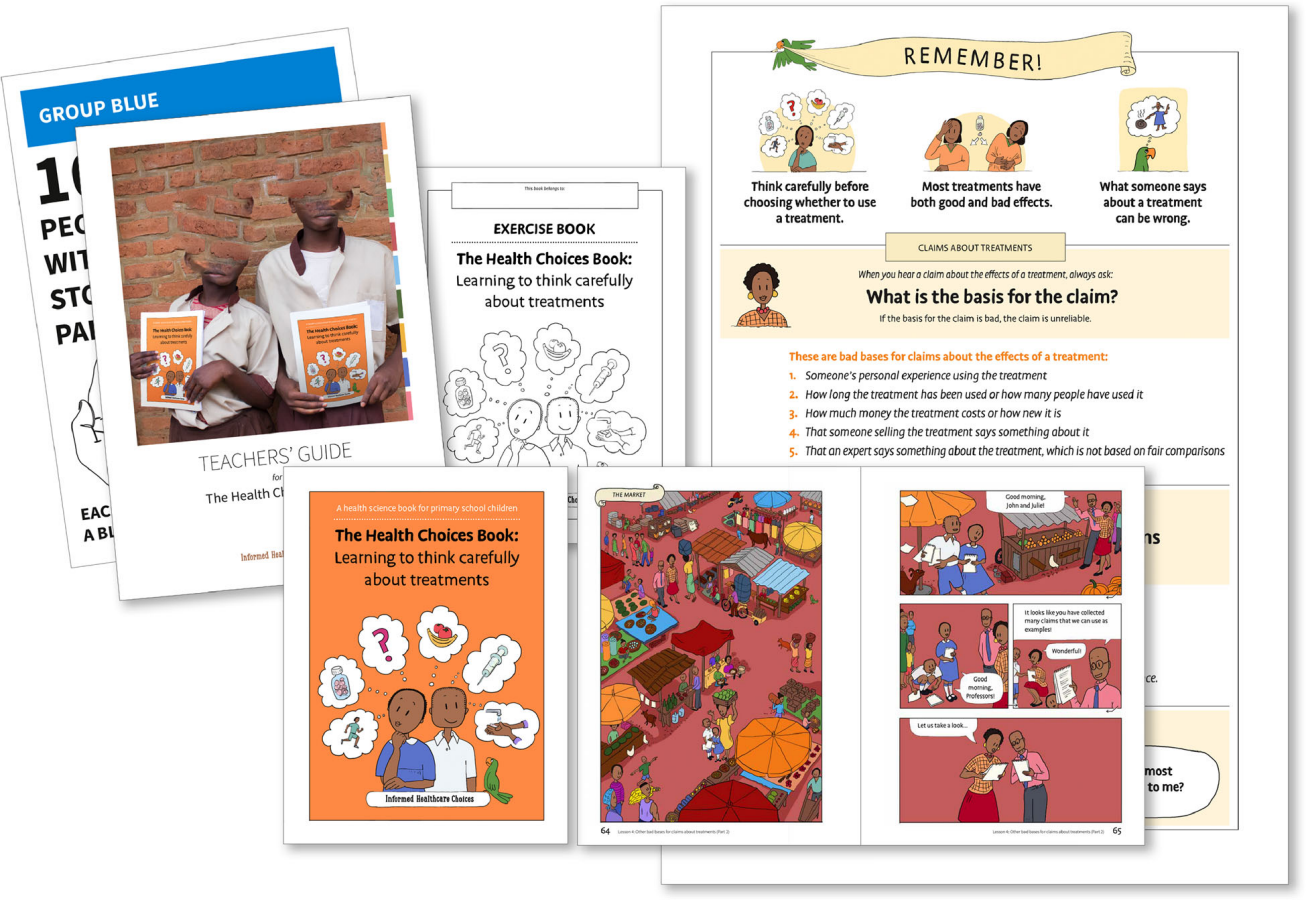
Version 3 (final) of the IHC primary school resources

We created Version 3 of the children’s book with 10 chapters, and a teachers’ guide (Fig. 5). We also created a separate exercise book, a classroom poster of the key learning objectives (the 12 Key Concepts), and a set of activity cards for one of the chapters. These open access resources can be viewed or downloaded at http://www.informedhealthchoices.org/primary-school-resources/. The contents of the children’s book and the teachers’ guide are summarised in Table 5.

**Table 5:** Contents of the children’s book and the teachers’ guide

Children’s book *The Health Choices Book: Learning to Think Carefully about Treatments. A health science book for primary school children* *Introduction* Lesson 1: Health, treatments and effects of treatments *John and Julie learn about CLAIMS about treatments* Lesson 2: Someone’s experience using a treatment Lesson 3: Other bad bases for claims about treatments (Part 1) Lesson 4: Other bad bases for claims about treatments (Part 2) *John and Julie learn about COMPARISONS of treatments* Lesson 5: Comparisons of treatments Lesson 6: Fair comparisons of treatments Lesson 7: Big enough fair comparisons of treatments *John and Julie learn about CHOICES about treatments* Lesson 8: Advantages and disadvantages of a treatment *Review* Lesson 9: Review of what is most important to remember from this book Glossary	Teachers’ Guide *Teacher’ guide for the Health Choices Book* The teacher’s guide includes an introduction to the project and the resources, and the following for each lesson, in addition to the embedded chapter from the children’s book: • The objective of the lesson • A lesson preparation plan • A lesson plan • A list of materials that the teacher and children will need • A synopsis of the story • Keywords in the chapter • Review questions to ask the children after reading the story • Extra examples for illustrating the concepts • Background about examples used in the story • Teacher instructions for the classroom activity • Answers and explanations for the activity • Answers and explanations for the exercises • Background information, examples, and keyword definitions for teachers

## Discussion

While focussing on the six facets, (usefulness, ease of use, understandability, credibility, desirability and identification), of Rosenbaum’s adaptation of Peter Moville’s honeycomb frame work of user experience, this work highlights the following lessons for future studies designing educational materials;

### Usefulness

Findings from the idea generation and exploratory prototypes phase of the project highlighted the need to clarify the usefulness of the resources for both teachers and children. Teachers’ participating in the Uganda teachers’ network workshop initially assumed that the purpose of the resources was to convey public health messages about the benefits of specific interventions, such as handwashing, healthy eating habits and exercise. Many of the ideas and prototypes generated at that workshop focused on communicating typical public health messages, rather than teaching children to think critically about health claims and choices.

There are several plausible explanations for this. These include that teaching is largely didactic in East Africa, in part due to large student-to-teacher ratios. This makes it difficult to use more interactive teaching strategies required to teach critical thinking skills [33]. Teaching critical thinking skills has not been a priority in primary school curricula or for evaluations of interventions to improve primary school education [12, 34, 35]. Previous public health interventions in schools have also tended to focus on promoting specific behaviours, rather than teaching children to think critically. This contrasts with our findings in Norway. Critical thinking was a priority for older children (in the International Baccalaureate IB programme) at the international school where we piloted the second version of the resources. However, the teachers there found that students entering the IB programme were not sufficiently prepared. They wanted to test our resources specifically to find out if they might help to address this problem that they had already identified.

Expectations of the children in response to early prototypes were different from those of the teachers. They assumed that the purpose of the resources was to help them do better in science and to learn to become scientists or health professionals.

We addressed these misunderstandings about why the resources are useful in several ways. We added introductions to both the children’s book and the teachers’ guide clarifying the purpose of the resources. These went through several iterations and we obtained feedback from teachers and children to ensure that the introductions clarified the purpose of the resources and why they are useful. We ensured that the examples we used would not be misunderstood and that they clearly illustrated how each Key Concept could be used to assess relevant claims and to make informed choices. We modified the structure of the book, and subsequently organised the Key Concepts (from six groups to three groups), to clarify and reinforce the purpose and usefulness of understanding and applying them.

When testing the first and second versions of the resources we found that teachers and most children found the resources useful and correctly understood their purpose by the end of the lessons. In addition to the above changes, we also developed a workshop for teachers to introduce them to the resources and to help ensure that they started out with a clear understanding of the purpose of the resources. The workshop is described in detail in another article [36].

### Ease of use

We found that our initial ideas and prototypes were difficult to use, even in well-resourced schools with low student-to-teacher ratios. We also found that many of the Key Concepts were not well understood by the teachers. Frequently they went off script, making unsubstantiated claims themselves rather than helping the children learn how to assess claims. Using a comic book to introduce the Key Concepts solved the problem of ensuring that they were introduced and explained correctly. The illustrations facilitated engagement, understanding and made it easier for the children to read the text. This is consistent with previous research, which has shown that adding pictures to written language can increase attention, comprehension, and recall [32]. However, pictures can also be misunderstood and the feedback we received on the illustrations resulted in many changes - both specific and general. For example, feedback from several children resulted in changes to how Julie, one of the two children who are main characters in the comic book was portrayed. As one child remarked when asked about the drawings in an early version: “Julie looks like a rumour monger.”

We also discovered important changes that were needed to make the comic book usable in Uganda. Many of the children were not familiar with reading comics and were confused about the order in which the frames should be read. They also were not familiar with speech and thought bubbles. To address this problem, we added arrows to the comic, showing the order in which frames should be read and explained speech and thought bubbles in the introduction.

Using a comic book to introduce the Key Concepts functioned well both in East Africa, where it is common for classes to read aloud and in Norway, where role playing was used when reading aloud in class. Based on our observations and interviews, we concluded that there were several ways of reading the book. Rather than recommending one of these, we provided the advice based on what we had observed.

Our observations and feedback from the teachers resulted in several changes to the teachers’ guide to ensure that teachers found it useful. One change was to incorporate the children’s book in the teachers’ guide. This facilitated using the guide, which includes instructions and suggestions for the teachers, while reading the comic together with the children or doing the activities. Others included providing lesson plans, explanations written for the teachers, and extra examples that the teachers could use to illustrate the Key Concepts.

The most important problem that we found with the second version of the resources was insufficient time. Teachers struggled to get though the lessons in 40 min and, therefore, the children were often confused and had not learned some of the Key Concepts. To address this problem, we reduced the number of concepts that were included from 24 to 12 and we doubled the amount of time for each lesson. This required us to step back and acknowledge that we had made a classic mistake of trying to teach everything about a topic at once, thereby overloading both the children and the teachers with too much information. By recognising that the resources are just one cycle in a spiral curriculum [37], we could make this dramatic change. Resources for subsequent cycles can build on what was previously learned from these resources and reinforcing while introducing new concepts.

Other changes that we made to the resources to improve their usability included greatly simplifying the activities to ensure that they could easily be managed by a single teacher with many children and ensuring that the exercises could be done by the children without placing a substantial burden on the teacher.

### Understandability

**Fig. 6:**
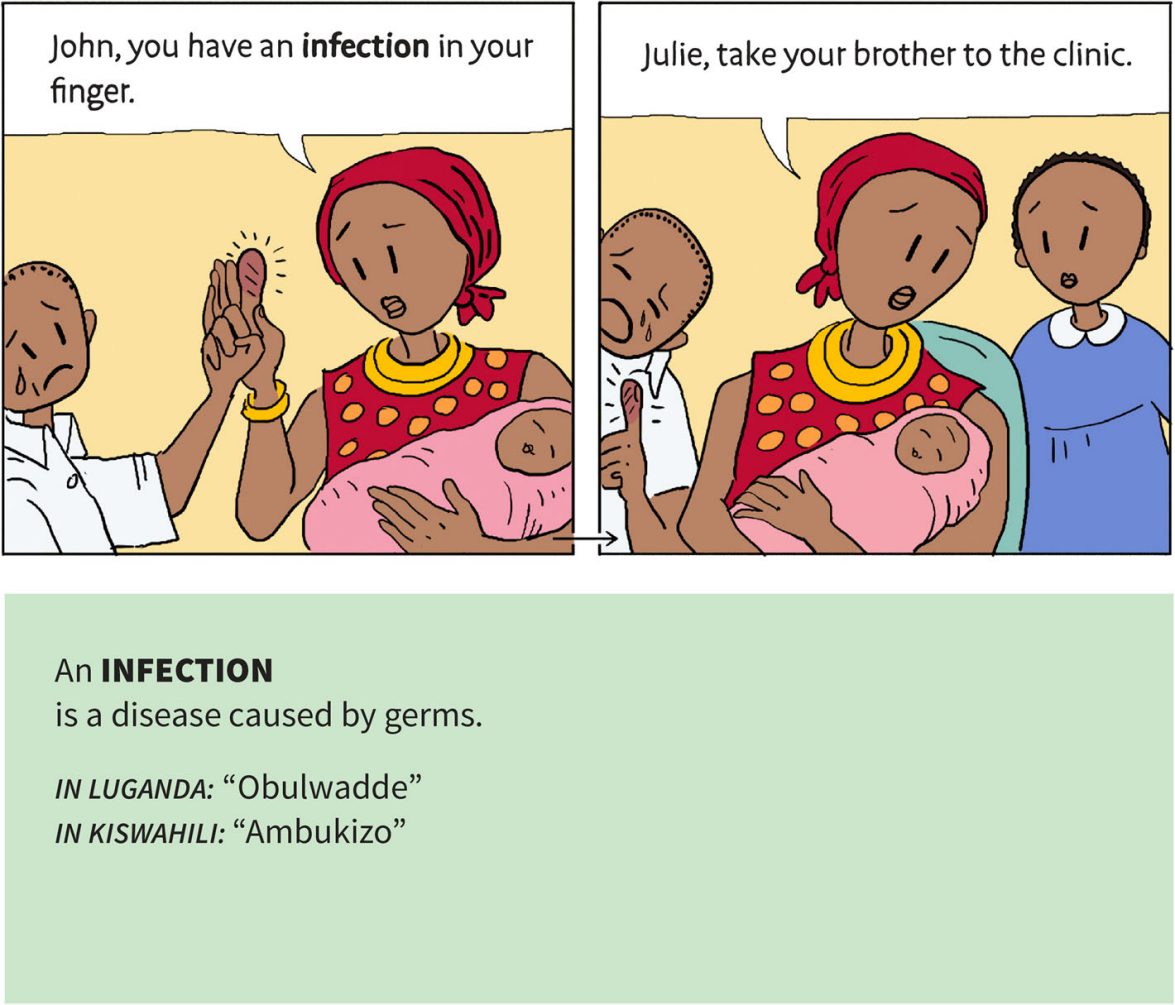
Repeating keywords where they first appear in the text

We discussed understanding the purpose of the resources in relation to its perceived usefulness and how that affected the extent to which teachers and children valued the resources. We also found substantial problems with understanding of the content. Many of the children read poorly and English was a second language for most. We found that words that we assumed 10 to 12-year-old children would understand, such as ‘health’, were new words for many children in East Africa. Although using a comic book with illustrations helped to improve understanding, we still needed to further simplify the language that we used explain terms. We addressed this by iteratively testing and rewriting the text, adding a glossary, adding translations of key terms to Luganda and Kiswahili, adding a list of new keywords used in each chapter, and adding explanations and translations of key terms to the text on the page where they were first used (Fig. 6). Together with teachers and children, we also generated a list of terms that were difficult for the children. We avoided using those terms if there was a good alternative or explained them.

**Fig. 7:**
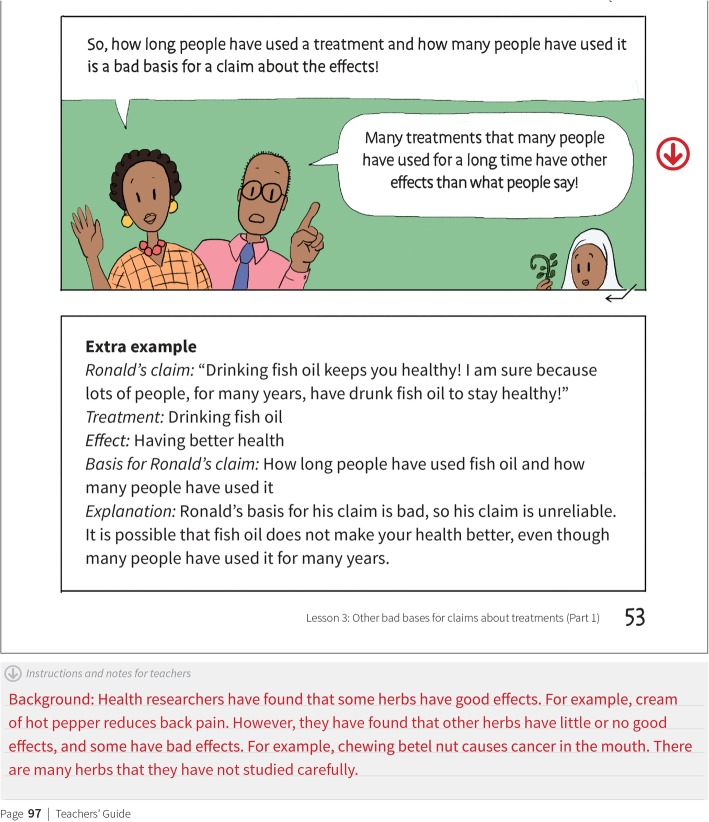
Background section of each chapter for teachers

Several changes to the teachers’ guide were made to ensure their understanding, these included adding a background section to each chapter and extra information about the examples that we used (Fig. 7), in addition to the workshop for teachers noted above.

### Credibility

Two problems that we identified were the use of magical elements in the first comic prototype and the inclusion of a talking parrot. We eliminated the former, but elected to keep the parrot for two reasons. First, although teachers were concerned that a talking animal would result in a loss of credibility amongst the children, none of the children perceived this as a problem. Second, the children responded very positively to the parrot, which both brought humour into the story and served as a source of claims. We did, however, review our use of the parrot to ensure that it was used consistently and that it was not included unnecessarily; e.g. repeating something that one of the other characters said.

### Desirability

Many of our early ideas, which focused on games, were clearly not something that the teachers wanted. They were difficult to organise and to manage, especially in classes with large student-to-teacher ratios.

We found that the book was highly desirable both in East Africa and in Norway. This was, perhaps, not surprising in East Africa where the schools had few books. However, the children at the international school in Norway also were very positive about the book. They uniformly responded that they would prefer the book to a computer game. It is uncertain to what extent this was because they had been exposed to poorly designed learning games or because the book was well designed. Children in both settings had not previously been exposed to use of a comic book to teach science.

The rationale for using a narrative in the book to explain the Key Concepts is that people often make sense of their lives through stories they hear and share with others [31]. Providing information in a story may resonate with people who might struggle to understand abstract concepts. Furthermore, characters in the narrative can role model new behaviours, enhancing self-efficacy [38]. Evaluations of the effects of narrative interventions support their use. For example, evaluations of the use of narratives in the context of health promotion have found that narrative interventions improve knowledge about health-promoting behaviours and the behaviours themselves [31].

Although we received consistent feedback from the children and teachers that they would prefer resources printed in colour, we also observed that the children clearly enjoyed colouring the prototype line drawings printed without colour. Another problem was that while we had hoped the children would take the books home and share what they were learning with their families, the teachers were worried about the books getting lost and the children not having them in class when they were needed. Our solution to both these problems was to create separate exercise books and textbooks. The final version of the children’s (text) book was in colour, could be kept at school, and could be re-used by other classes. The exercise book (containing key learning goals for each lesson, exercises and a glossary) was printed in black-and white that could be coloured by the children, and could be taken home.

### Identification

Initially we received many comments from the children in Uganda about the drawings, particularly about John and Julie, with whom they did not identify. However, with subsequent iterations of the children’s book, the children identified with John and Julie. Similarly, both the teachers and children expressed that the resources felt like they were appropriate for them, increasingly with each iteration.

We were uncertain to what extent children at the international school in Norway would find the characters and the story, which was set in an East African context, relevant to them. To our surprise, we found that some of the children when asked where they thought the setting for the story was did not notice that it was in Africa. Others we spoke to were pleased that the story was set in Africa rather than in North America or Europe, which was the setting for most of the books they used.

## Conclusions

Our findings suggest that with the iterative revisions of the IHC primary school resources, users - both children and teachers - experienced the resources as useful, easy to use, understandable, credible, desirable, and well suited for them. We believe there are two closely related reasons why we could achieve this. First, our grant application did not include a specification of what we were going to create. Instead, we described our goals and the methods that we would use to develop resources. This allowed us ample time (two years) to generate and prototype ideas and then to iteratively design, pilot and user-test, analyse, and redesign these resources.

Second, we used a user-centred design approach with a multidisciplinary team and engagement of users throughout the development process. The research team included health service researchers with diverse backgrounds, designers, and a journalist. We collaborated closely with a teachers’ network, a journalists’ network [39], policymakers, and education researchers. We also piloted and user-tested the resources in schools in four countries. This broad range of feedback helped us create resources that increasingly resonated with these diverse communities.

The main limitation to the study was time constraint, in terms of tying the design schedule to the already busy school schedule. This also meant that only schools that were willing to avail time and participated in the development may not have been representative of the larger population. However in a follow up study, we have evaluated the effects of using the resources in a large randomised trial in Uganda (with 120 schools), that showed the intervention led to large improvements in the ability of both the children and their teachers to assess claims about treatments [36]. This trial excluded schools that participated in the development. Therefore, we can be fairly sure that input we gathered from participating schools was also representative for larger groups.

## Supplementary information

**Additional file 1.** Overview of the development

**Additional file 2.** Structured Observation Form- Chapter one.

**Additional file 3.** Prioritisation of Key Concepts.

**Additional file 4.** Prototyping.

**Additional file 5.** User Experiences of the IHC resources.

## Data Availability

All data will be available on reasonable request. (Extra data can be accessed on https://www.informedhealthchoices.org/learning-resources/).

## Abbreviations

CARL: Critical thinking and Appraisal Resource Library
IB: International Baccalaureate Programme
IHC: Informed Health Choices Project
