# Supplementary material for: Development of the informed health choices resources in four countries to teach primary school children to assess claims about treatment effects: a qualitative study employing a user-centred approach
Source: Pilot Feasibility Stud. 2020 Feb 10;6:18. doi: 10.1186/s40814-020-00565-6 (PMC7008535; doi:10.1186/s40814-020-00565-6)
Supplement: Supplementary file 1 — Additional file 1. Overview of the development [file 40814_2020_565_MOESM1_ESM.docx]

**Supplementary file 8. Overview of development**

| ***Methods and dates*** | ***Participants*** | ***Description of activities*** | |
| --- | --- | --- | --- |
| **PRIORITISATION OF KEY CONCEPTS** | | | |
| **Prioritsation of Key concepts workshop**  Uganda  August 2013 | The teachers’ network in Uganda (24 teachers) and the research team (AN, DS, NS) | | Members of the teachers’ network attended a three day meeting where concepts were presented, discussed and prioritised using a pre-set criteria ([19](#_ENREF_19)). |
| **IDEA GENERATION AND EXPLORATORY PROTOTYPES** | | | |
| **Review of existing resources**  February 2013 to September 2014 | The research team (AA, AM, AN, AO, CG, DS, SL, SR) | We searched for and reviewed existing resources ([28-30](#_ENREF_28)). | |
| **Idea generation workshop**  *(Participatory collaboration)*  February 2013 | Researchers, teachers, and journalists from Indonesia, Nepal, Norway, Uganda, and the United Kingdom | At the 3-day kick-off meeting for the project, the research team together with invited teachers and journalists (18 people) discussed which concepts to focus on and brainstormed about potential resources. Journalists were participating as part of a parallel project to develop mass media resources. | |
| **Pilot testing in Norway**  *(Participatory observation)*  February 2013 | Approximately 30 ten-year old children and 2 teachers at an international school in Norway, and the research team (AA, AM, AN, AO, CG,JM, SP, SR, TT) | We developed materials and pilot tested an exploratory prototype (an experiment with colored candies)([28](#_ENREF_28)). | |
| **Prototyping workshop**  *(Facilitation & non-participatory observation)*  September 2013 | The teachers’ network in Uganda (24 teachers) and the research team (AA, AM, AN, AO, CG, DS, NS, SR.) | This was a full-day workshop at which teachers brainstormed and created prototypes([19](#_ENREF_19)). | |
| **Pilot testing in Uganda**  *(Participatory observation)*  September 2013 | 46 children and two teachers at a private school and 129 children and two teachers at a government school and the research team (AA, AM, AN, AO, CG, DS, MK, SR.) | We pilot tested the same exploratory prototype that was tested at the international school in Norway. | |
| **Meeting with the Ugandan National Advisory Board**  December 2013 | 15 members of the National Advisory Board for the project | This was a half day meeting during which ideas and barriers and facilitators to implementing these were discussed in the Ugandan context.  and facilitators that would hinder or facilitate the successful implementation of the project. | |
| **Analysis of findings and idea generation**  December 2013 to January 2014 | The research team (AA, AM, AN, AO, CG, DS, SL, SR) | We reviewed ideas that had been generated and their pros and cons. Based on this, we coded the ideas as ‘good’, ‘not sure’, or ‘drop’, and we identified principles to guide development of the resources. | |
| **Pilot testing in Uganda and Norway**  *(Participatory observation)*  April 2014 | 27 nine to 13-year-old children and two teachers at 1 private school in Uganda and approximately 30 10-year-old children and 4 teachers at an international school in Norway, and the research team (AA, AO, AN, DS, MO, SR) | We developed and piloted a game designed to teach what a “testable question” is, using charts with relevant examples. The game included instructions, score sheets, timers, and a question bank. We also tested an activity where the children designed and carried out an experiment using paper airplanes. | |
| **Prototyping and pilot testing in Norway**  *(Participatory observation)*  May to September 2014 | Four 12-year-old girls, and the research team (AA, AM, AO, CG, SL, SR) | We prototyped a series of eight games and piloted these. We first tested each game by playing it ourselves. At each meeting with the children we introduced the relevant Key Concepts using a PowerPoint presentation, then played the game, then collected feedback. | |
| **Meeting and discussions with the members of the teachers’ network**  September 2014 | 24 members of the teachers’ network and 2 teachers who participated in the piloting of the materials, and the research team (AN, DS) | We updated the teachers about the prototypes we had user-tested and piloted. We asked them to try out the games in small groups of three before giving us feedback on what they thought about the progress being made so far. We also asked two teachers who had participated in the piloting of the materials to share their experiences with the rest of the group. The teachers discussed the challenges they faced and how they handled them. | |
| **Analysis of findings and idea generation**  September 2014 | The research team (AA, AM, AN, AO, CG, DS, MO, SL, SR) | We reviewed our experience from prototyping and piloting the series of games. Based on this, we decided to develop a children’s book to introduce the Key Concepts using a comic story and a teachers’ guide. | |
| **Development of a partial prototype**  September to October 2014 | The research team (AA, AM, AN, AO, CG, DS, MO, SL, SR) | We developed two chapters of the children’s book and teachers’ guide, including activities MO prepared a manuscript for each chapter, which was converted to a comic book by SR with exercises and activities. MO prepared a draft of each chapter of an accompanying teachers’ guide and AM designed the guide. Each chapter was reviewed by the rest of the research team and went through three iterations. | |
| **Pilot testing** *(non-participatory observation)*  **and user-testing in Uganda**  October 2014 | 73 year-5 children and two teachers at a government school and 28 year-5 children and one teacher at a private school piloted the prototype, and the research team (AM, AN, AO, DS, MO, SR) | We pilot tested the first chapter of the children’s book and teachers’ guide, including activities, at both schools and the second chapter at the private school. We interviewed four 10 to 15-year-old children (two from each school) and all three teachers. | |
| **Analysis and idea generation**  November 2014 | The research team (AA, AM, AN, AO, CG, DS, MO, SL, SR) | We coded findings from the pilot and user testing as “show stoppers” (very important problems), important problems, minor problems, positive feedback, or specific suggestions. | |
| **Feedback gathering meetings**  December 2014 to January 2015 | National Advisory Board for the project and the teachers’ network | AN, DS, and NS presented plans for a complete prototype of the IHC primary school resources and sought feedback and input. We conducted a half-day meeting with the National Advisory Board and a full-day meeting with the teachers. AN, DS and NKS recorded the sessions as the policymakers and stakeholders discussed. | |
| **VERSION 1 OF THE IHC PRIMARY SCHOOL RESOURCES** | | | |
| **Development of a complete prototype**  December 2014 to April 2015 | The research team (AA, AM, AN, AO, CG, DS, MO, SL, SR) | We outlined a children’s book with 10 chapters that would cover 24 Key Concepts. MO prepared a storyboard for each chapter, which was converted to a comic book by SR with exercises and activities. MO prepared a draft of each chapter of an accompanying teachers’ guide and AM designed the guide. Each chapter was reviewed by the research team and went through three iterations. | |
| **Pilot testing** *(non-participatory observation)*  **and user-testing in Uganda**  January to May 2015 | 67 year-5 e children and one teacher at a government school, and 32 year-5 children and one teacher at a private school, and the research team (AN, DS, research assistents) | We gave the teachers a chapter a week before they taught each lesson. The teachers who taught the lessons were not given any instruction other than what was in the teachers’ guide. AN and DS observed each lesson. They recorded their observations using a semi-structured guide. They interviewed three children from each school and both teachers after each chapter was pilot tested using a semi-structured interview guide. There was an observer who took notes at each interview. All interviews were audio recorded. | |
| **Feedback gathering meeting with the teachers’ network**  May 2015 | 24 members of the teachers’ network, 2 teachers that had participated in the piloting of the materials, and 2 teachers that had participated in the user testing of the materials | This was a one day meeting where we grouped the teachers in small groups of about three teachers per group and asked them to read a chapter and give us feedback on what needed to be improved and what should be dropped. AN, DS, and NKS recorded the sessions as teachers gave their feeback. | |
| **Analysis and idea generation**  May 2015 | The research team (AA, AM, AN, AO, CG, DS, IC, MK, MO, NS, SL, SR) | AN and DS entered the findings in a spreadsheet. For each finding, AN, AO, DS, MO, and SR coded its importance (very important, important, or less important); whether it was a problem, an idea, or positive feedback; and whether it applied to the entire book, a specific chapter, or was a repeat of a previous finding. The findings were summarized for the research team and the major findings and plans for the second version were discussed and agreed. | |
| **VERSION 2 OF THE IHC PRIMARY SCHOOL RESOURCES** | | | |
| **Development of the second complete prototype**  June to August 2015 | The research team (AA, AM, AN, AO, CG, DS, IC, MK, MO, NS, SL, SR) | MO prepared a revised draft for each chapter, SR revised the drawings and AM revised the design of the guide. Each chapter was reviewed by the research team and went through three iterations. | |
| **Pilot testing** *(non-participatory observation)*  **and user-testing in Uganda**  September to December 2015 | 96 year-5 children and one teacher at a government school and 109 children and one teacher at another government school, and the research team (AN, DS, research assistents) | We gave the teachers the materials in two parts (chapters 1 to 4 and chapters 5 to 10). The teachers who taught the lessons were not given any instruction other than what was in the teachers’ guide. AN and DS observed each lesson. They recorded their observations using a semi-structured guide. They reviewed the children’s completed exercises from the previous lesson and they interviewed two children from each school and the two teachers after each chapter was pilot tested using a semi-structured interview guide. They also interviewed an additional teacher who did not teach the lessons. There was an observer who took notes at each interview. All interviews were audio recorded. | |
| **Pilot testing** *(non-participatory observation)* **and user-testing in Kenya**  September to December 2015 | 30 children between 10 to 14 years old and one teacher, and the research team (MK and research assistents) | The teachers was initially provided with the teachers’ guide prior to the pilot and user testing to enable him familiarize himself with the materials. The relevant lesson for each week was covered by the children under the guidance of the teacher. Following each lesson, between 4 and 6 pupils were interviewed using a semi-structured guide. An observer recorded the interviews with the children. A semi-structured guide was used to report the lesson findings. Some of the lessons and interviews with pupils were recorded. | |
| **Pilot testing** *(non-participatory observation)* **and user-testing in Rwanda**  September to December 2015 | 33 year-5 children (10 to 12-years old in year-5) and one teacher, and the research team (MM and AU) | MM and AMU used the same methods for the pilot study as described above for Uganda and Kenya. Two or three observed 10 class sessions (non participatory observation). Then after each lesson, one of them interviewed and the other taken notes for three of the children in a focus group and the teacher (individually) in an interview using a retrospective think aloud technique, going through each page of the book with a semi-structured interview guide. The interviews were audio recorded. | |
| **Pilot testing** *(non-participatory observation)* **and user-testing in Norway**  September to December 2015 | Three year-7 classes with 15 to 18 children from many different countries in each class and two teachers (one who taught two different classes) at an English-language international school and the research team (MO, SR, AO, AA) | One or two researchers observed each lesson in each class using a structured data-collection form and then entered findings into a spreadsheet. We interviewed four children selected by one of the teachers with one person conducting the interview and one observer; we interviewed each teacher twice, and we collected verbal feedback from each class after they completed all 10 chapters; we reviewed their completed exercises, and we interviewed the school’s head of science. All the interviews were semi-structured using interview guides and were recorded. | |
| **Update on current activities and feedback gathering meeting of teachers’ network members**  December 2015 | 24 members of the teachers’ network | At this full-day meeting, teachers were updated on the progress before being divided in groups of about three. We asked each group to look at the entire chapter of the teachers’ guide assigned to their group and provide the research team (AN, DS, and NKS) with feedback on what needed to be addressed. | |
| **Analysis and idea generation**  December 2015 to January 2016 | The research team (AA, AM, AN, AO, AU, CG, DS, IC, MK, MM, MO, NS, SL, SR) | For each finding, AN, AO, DS, MM, MO, and SR coded its importance (very important, important, or less important); whether it was a problem, an idea, or positive feedback; and whether it applied to the entire book, a specific chapter, or was a repeat of a previous finding. The findings were summarized for the research team and the major findings and plans for the second version were discussed and agreed. | |
| **VERSION 3 OF THE IHC PRIMARY SCHOOL RESOURCES** | | | |
| **Development of the final set of learning resources**  January to March 2016 | The research team (AA, AM, AN, AO, AU, CG, DS, IC, MK, MM, MO, NS, SL, SR) | MO prepared a storyboard for each chapter with exercises and activities. This was converted to a comic book by SR. MO prepared a draft of each chapter of an accompanying teachers’ guide and AM designed the guide. Each chapter was reviewed by the research team and went through three iterations. | |
