# Supplementary material for: Development of the informed health choices resources in four countries to teach primary school children to assess claims about treatment effects: a qualitative study employing a user-centred approach
Source: Pilot Feasibility Stud. 2020 Feb 10;6:18. doi: 10.1186/s40814-020-00565-6 (PMC7008535; doi:10.1186/s40814-020-00565-6)
Supplement: Supplementary file 2 — Additional file 2. Structured Observation Form- Chapter one. [file 40814_2020_565_MOESM2_ESM.pdf]

IHC

PILOT OBSERVATION FORM

SCHOOL RESOURCES

CHPT.1

**SECTION A:**	**Pre-lesson**
**Observer:**	AN
**School:**	(X) primary school
**Scheduled start time of lesson:**	2:00 pm
**Scheduled end time of lesson:**	2:40 pm
**Number of children:**	100
**Age range of children:**<br>*From youngest to oldest child*	10 years (youngest) 15 years (oldest)
**Number of benches:**<br>*This is so we know about how much space each child had.*	24 benches
**Number of teachers in the room:**	one
**SECTION B:**	**Start of lesson**
**Actual start time of lesson:**	2: 13 pm
**What did the teacher do before the class started reading the chapter? For how long?**<br>*E.g. makes jokes, ask the children questions or give his own summary of the book or a chapter.*	- The teacher asked the children; to welcome the visitors<br>- The teacher also asked the visitors to introduce themselves.<br>- The teacher started by asking the children to name the body parts.<br>- The teacher introduced the book by writing the title on the black board.<br>- The teacher started introducing the words “treatment” and Effects. Doing it in both Luganda and English i.e. Treatment is obujanjabi. Giving an example of how we treat malaria. The children named malaria treatment like Quartum and then discussed effects i.e. ekivamu in luganda. The children named some effects like sweating, vomiting etc. The teacher also named some good effects for example getting better.
**SECTION C:**	**Reading**

	Page 5; the children struggled with the word injuries on page 5 in the children's book.<br>"Injuries" – Children asked the teacher. Teacher replied in Luganda "Injury is okukosebwa"
**Page 12--Chapter title page**	The teacher skipped reading the title "Claims, Comparisons and Choices; A health science book".
**Page 13--"Health"**	The children were able to read the word health and the teacher compared the two pictures on the page; i.e. "One child is healthy and doing back summer", and the other child is unwell.
**Page 14 to 15--"Treatments"**	- The children were able to read the word treatment.<br>- They struggled with reading the word "crutches". Teacher had to explain what crutches meant in Luganda and what it is used for.<br>- The word vegetable; some children struggled with that word
**Page 16 to 19--"Effects"**	Effects;<br>Teacher asked the children to first name treatments that they have read in the book; the children mentioned; using an equipment<br>The children started reading again; they read effects well and then continued reading the book.<br>The children named the good and bad out of playing football; i.e. when you play football…(children were asked to name them)<br>Good Bad<br>-Being strong -Getting a fracture
**Page 20 to 21--"things people say"**	The children were able to read the word herbs without difficulty.<br>Teacher kept referring to both words; i.e. treatment is obujanjabi
**Page 22 to 24--"This book"**	The children kept reading while the teacher would stop and interpret difficult words into Luganda for the children.
**SECTION D:**	**Activity**
	Teacher asked children;

	To discuss the examples of treatment; i.e. children mentioned exercising, eating fruit and vegetables, getting an operation, dressing a wound; using plasters on a broken arm.
**SECTION E:**	**Exercises**
	The children started doing their exercises in their books. Exercise 3; Children were a bit unsure; the teacher asked them to choose the most correct answer and write it down. The teacher kept explaining what was required and asked the children if they had understood the instructions to which the children replied yes they had.
**SECTION F:**	**Post-lesson**
**Who read aloud?**	All the children read aloud
**Actual end time of class:** *When did the teacher leave the children to work on their own or do other things?*	One hour; 3:00 pm
**About how long did the class spend reading the story?**	30 minutes
**About how long did the class spend doing the activity?**	10 minutes
**About how long did the class spend doing exercises with the teacher in the room?**	20 minutes
**About how long did the children break for?**	0 minutes
**Did the children seem interested or disinterested in the lesson? How so?**	The children were very excited, partly because they each got their own book.
**How did the teacher engage the children?**	The teacher encouraged the children especially the back benchers to answer questions as they arose.
**How did the teacher use the blackboard?**	The teacher kept writing key words on the blackboard.

**What did the children do with the books at the end of the class?** *Did they take them home or hand them in?*	The teacher collected the books and told the children that she would be marking them later.
