# Supplementary material for: Development of the informed health choices resources in four countries to teach primary school children to assess claims about treatment effects: a qualitative study employing a user-centred approach
Source: Pilot Feasibility Stud. 2020 Feb 10;6:18. doi: 10.1186/s40814-020-00565-6 (PMC7008535; doi:10.1186/s40814-020-00565-6)
Supplement: Supplementary file 3 — Additional file 3. Prioritisation of Key Concepts. [file 40814_2020_565_MOESM3_ESM.docx]

**Supplementary file 10. Prioritising Key Concepts**

We started with the list of 32 Key Concepts identified at the beginning of this project ([15](#_ENREF_15)), organised in six groups:

- Recognising the need for fair comparisons of treatments
- Judging whether a comparison of treatments is a fair comparison
- Understanding the role of chance
- Considering all the relevant fair comparisons
- Understanding the results of fair comparisons of treatments
- Judging whether fair comparisons of treatments are relevant

To determine how many of these Key Concepts the prototypes should teach, we first consulted with the teachers’ network at a workshop in August 2013. They found all six groups of concepts to be relevant for year-5 (10 to 12-year-old) children ([19](#_ENREF_19)). Based on input from the teachers, we judged that primary school children could learn 24 of the 32 concepts. Members of the research team made these judgements in a face-to-face meeting in September 2013, using informal discussion to reach a consensus.

However, 24 concepts proved to be too much to learn in a school term. The early prototypes we created had too many concepts per lesson and took too long to teach in a normal school hour (40 minutes). We also observed that the teachers needed time to repeat material from previous lessons. After each round of proto typing, we eliminated more Key Concepts from our list.

We decided which ones to eliminate by considering the importance of the concepts and the difficulty that the children had learning them. The importance of the concepts was based on judgements made by members of the research team by:

- Each person individually identifying which of the 24 Key Concepts they considered most important
- Compilation and discussion of those judgements
- Voting on the concepts
- Reaching a consensus by informal discussion

At a face-to-face meeting in May 2015, we reached agreement that eight of the concepts were most important for our target population in Uganda. Three members of the research team also reviewed data from our piloting and user-testing and identified concepts that appeared to be too difficult to teach to 10 to 12-year-old children. Later, based on feedback from piloting the resources, we considered how the concepts were grouped in the lessons and the number of concepts being taught in each lesson.

We ended up using 12 of the 24 concepts in the final version of the resources (Box 1) and reorganised them into three groups to simplify and clarify their purpose:

- CLAIMS: “questions you should ask when someone says something about a treatment”
- COMPARISONS: “questions that health researchers ask to find out more about the effects of treatments”
- CHOICES: “questions that you should ask when you are choosing whether to use a treatment”
