# Supplementary material for: Development of the informed health choices resources in four countries to teach primary school children to assess claims about treatment effects: a qualitative study employing a user-centred approach
Source: Pilot Feasibility Stud. 2020 Feb 10;6:18. doi: 10.1186/s40814-020-00565-6 (PMC7008535; doi:10.1186/s40814-020-00565-6)
Supplement: Supplementary file 4 — Additional file 4. Prototyping. [file 40814_2020_565_MOESM4_ESM.docx]

### Supplementary file 11. Prototyping and pilot testing in Uganda and Norway

### Prototyping workshop with teachers’ network in Uganda

All 24 members of the teachers’ network attended this full-day workshop, facilitated by eight members of the IHC team (Figure 1). We guided the teachers through a structured process that led them to create a persona, identify barriers and facilitators, generate ideas about resources, and rapidly prototype some of the ideas. Persona are fictional characters created to represent a user type, often used in digital design development ([31](#_ENREF_31)).

***Figure 1. Prototyping workshop with teachers’ network in Uganda***


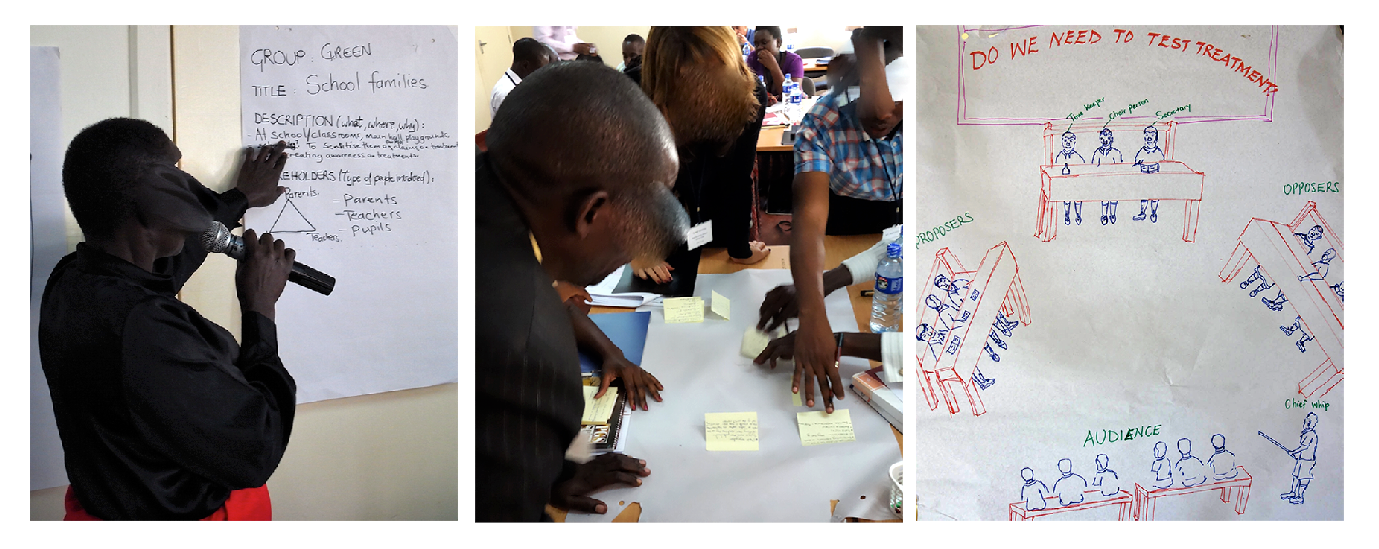


Some of the categories of ideas teachers came up with were: assemblies, community involvement, family involvement, debates, use of drama, games, experiments, reading materials, video, teacher education, and curriculum development.

Although the resulting prototypes did not represent resource ideas we could use directly (as the ideas depended too much on teacher’s in-depth prior knowledge gained through their participation in our workshop), we developed a better understanding of teachers’ perspective on our work as well as many insights into the school and community setting in Uganda. Key messages were that:

- There was a paucity of available materials in schools, including paper and printers.
- Songs, drama, and storytelling were popular methods for conveying health messages.
- Children might be expected to share their knowledge with their family or community.

We found that it was difficult for some people to grasp that we were focusing on teaching health literacy (specifically critical thinking about treatment claims) and not health promotion (teaching about the benefits of specific “treatments” such as handwashing or using insecticide treated bed nets).

Drawing on what we learned, we focused our next efforts on creating prototypes of highly structured materials that we could produce and provide cheaply for each school, and that did not rely on teachers’ prior knowledge about the Key Concepts. We continued to try to create games.

### Prototyping and pilot testing in Uganda and Norway

We developed and piloted a game designed to teach what a “testable question” is and tested an outdoor activity where the children designed and carried out an experiment using paper airplanes. We piloted and user-tested these games at a Ugandan school and at an international school in Norway (Figure 2).

***Figure 2. Pilot testing the prototype of a “testable question” game in Norway***


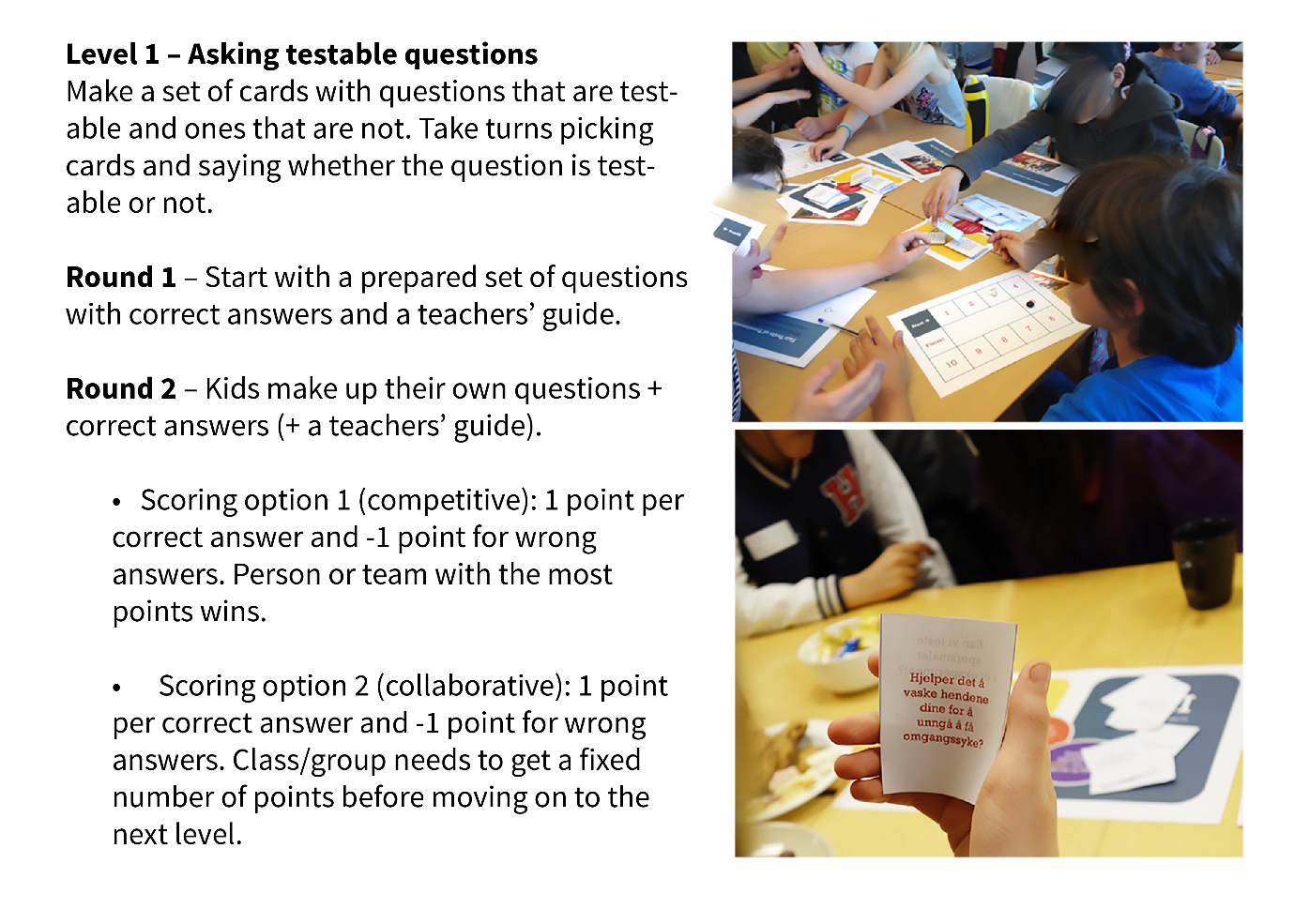


Then we developed eight prototypes for simple games with increasing difficulty, designed to teach one concept each. These were based on materials that could be printed on paper. We tested these out in a series of meetings over two months with a group of four 12-year-old girls in Norway. At each meeting with the children we introduced the relevant Key Concepts using a PowerPoint presentation, then played the game, then collected feedback.

### Development of a partial prototype of a comic book and teachers’ guide

We developed two chapters of a children’s comic book and teachers’ guide, with an activity description in each and a separate “activity booklet”. We carried out pilots (with non-participatory observation) and user-tests in two Ugandan schools as described in Additional file 1, followed by data analysis (Figure 6).

***Figure 3. Pilot testing a partial prototype of comic book and teacher’s guide***


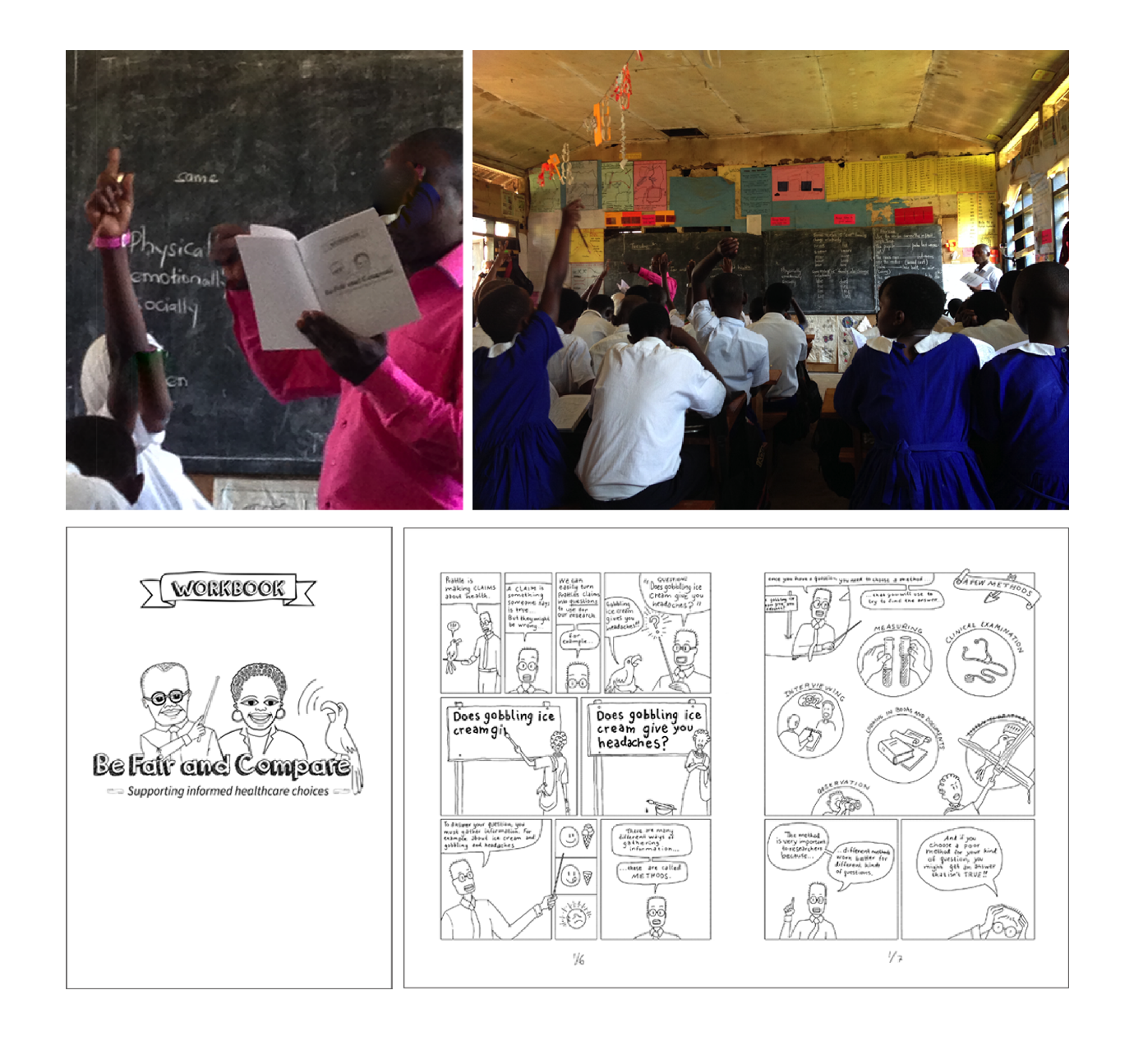


### Negative user experiences

We categorized several findings as very important negative findings (“show stoppers”). For example, in one of the schools, the class spent three and a half hours on the first chapter alone, with the teacher often improvising off-script and coming up with examples that led the children to ask many unrelated and misleading questions; for example:

“Does using the pit latrine while pregnant cause some mothers to lose their children, true or false?”

At the second school, the outdoor activity became very chaotic and took too long, leaving no time for discussion about what the children had learned. Also, some of the key terminology (e.g. ‘treatment’ and ‘outcome’) was too complicated for the children.

Other important negative findings included: The size of the text was too small in both books. Some children were reading a comic book for the first time and had difficulty following the flow of text and understanding the comic layout. We observed that some of the examples we used misled the children, for example:

“[I’ve learned] not to eat so much if you want to run fast”.

Other negative findings included some of the words we had used that were unfamiliar to both teachers and children (e.g. ‘Prattle’ and ‘gobbling’). Typical comic book sound words like ‘bling’ and ‘whoosh’ were also unfamiliar. In addition, some of the children did not want to identify with some of the characters, because they were not like them; for example:

“The girl has not combed her hair”. “John’s shirt is not tucked in”.

When asked about the usability of the product, the teacher said:

“First experience was difficult, will be better next time.”

### Positive user experiences

A very important positive finding was the fact that both teachers and children were enthusiastic about the use of comics. They liked the pictures and the way the information was presented which broke the routine of blackboard teaching.

“We didn’t just talk and use the chalkboard, there was a book for each child.”

When we asked teachers about the desirability of the product and its suitability for their pupils, there were several positive findings:

“The way the children were thinking, it was extraordinary.”

“The level is good for these children.”

However, it became clear to us from classroom observations and user-testing interviews, that the children had obviously not understood some of the content in the book; for example:

‘’The question I have about what John and Julie learn in this chapter is: Why did John and Julie use a coin to divide ten and a hundred people?" Why didn't they use counting to divide the people?”

Many children coloured in their books. The use of animals (e.g. “Prattle”, the parrot) seemed to be interesting to them, but there was disagreement among teachers and children about the appropriateness of including animals that could talk in the story. It was “not real” as stated by some teachers:

“What is the talking bird doing here?”

“The children will not take this seriously because of this bird here, they will think it is a joke.”

The children, however, liked Prattle; as one stated during a user-testing interview:

“I think that bird is so funny because it is saying many funny claims.”

Suggestions from teachers categorized as very important constructive findings included: providing extra examples, adding numbers to the frames so that the children know which direction to read, having a thicker paper on the cover of the teachers’ guide, colouring the pictures, and breaking the content into smaller teachable units.
