# Supplementary material for: Development of the informed health choices resources in four countries to teach primary school children to assess claims about treatment effects: a qualitative study employing a user-centred approach
Source: Pilot Feasibility Stud. 2020 Feb 10;6:18. doi: 10.1186/s40814-020-00565-6 (PMC7008535; doi:10.1186/s40814-020-00565-6)
Supplement: Supplementary file 5 — Additional file 5. User Experiences of the IHC resources. [file 40814_2020_565_MOESM5_ESM.docx]

### Supplementary file 12. User experiences of the IHC primary school resources

## Version 1 of the IHC primary school resources

### Negative user experiences

The objective of the lessons and materials was misunderstood, as we found out in our interviews with some of the children. One of the very important negative findings was an incorrect understanding of what the book was about. Some children expected they were going to evaluate treatments themselves or learn about practicing medicine.

“I am going to learn being a scientist and being a health treater and treating other people”

“I think I will learn more about science and health. The needs of being a doctor and how I will become one”.

Two chapters were too long to be completed in one school hour (lesson), and a teacher said there were too many lesson goals. There was much concept-related vocabulary that some children did not understand despite the definitions and translations; e.g. claim, outcome, substitute outcome, assumption, unreliable, careful summary. Additionally, some children struggled with simpler English words; e.g.: expert, also, normally, reward. Feedback suggested that English skills would likely be even poorer in more rurally located schools. One child stated during an interview:

“This book is for a school like ours which knows hard English, but not for the village schools”.

Another important negative finding in this version was the volume of the teachers’ guide and the content in the children’s book. Some teachers suggested that the chapters should be split into manageable units. One of the pilot teachers attending the network meeting stated:

“I found the information in the chapter to be too much when delivering the lesson, it was difficult to have the children read the chapter and also do all the exercises in the 45 minutes of the lesson.”

A member of the teachers’ network said:

“This lesson plan is difficult to follow.”

Other important negative findings for this version included the use of the English language, which the target audience (teachers and children) still found difficult to understand. One of the children said in an interview:

“The difficult word was ingredients. I don’t know what it means.”

In addition, teachers were not comfortable with some of the English words used in the teachers’ guide and in the children’s book. One stated:

“The children’s book had some confusing and difficult words like ‘effects’. Yet, in my own understanding, I had a different meaning of the word effect.”

Other negative findings included that the illustrations (which were still just sketches drawn with pencil) were unclear and the hand written text was difficult for many to read. Some of the children were unsure about how to fill in the answers to the exercises and others struggled to read the comic in the right direction, despite the simplified layout.

In addition, we also observed teachers improvising from the text and offering incorrect examples and analogies. Teachers expressed wanting step-by-step instructions that could also be followed by a stand-in teacher who was unfamiliar with the project and more in-depth information to help them be prepared if challenged by children.

One teacher suggested making the professor characters in the story doctors, and others suggested changing their names.

### Positive user experiences

The teachers felt this version was a big improvement over the partial prototype, and that the content became clearer from chapter to chapter.

Using a combination of pictures and text seemed to be helpful both from the teachers’ and children’s perspective. One child stated:

“The words help you understand the pictures.”

Another important positive finding was that the exercises seemed to be the right level of difficulty. Both teachers and children valued them and suggested that more exercises should be included. One of the teachers said this about the exercises in the book:

“Good, perfect, children will be able to do this and it even helps them improve on the area of English.”

The exercises in the book seemed tuned to the children’s literacy levels with many children correctly completing the exercises within 5-10 minutes. The Luganda translation was also perceived as helpful. One child said:

“Effect - it means Ekivaamu. It is good to tell us the meaning of the word.”

Children loved the parrot. One of them suggested:

*“More pictures about birds.”*

## Version 2 of the IHC primary school resources

### Version 2 - Negative findings

Some of the very important negative findings for this version included: feedback from the pilot teachers who felt that some chapters in this version were very long, particularly chapter 8. One pilot teacher stated:

“Some of these chapters, like chapter 8, need to be divided into smaller chapters. They take a lot of time.”

A member of the teachers’ network said:

“The content is too wide”.

Another very important negative finding arose from observations. In a class of about 109 children, a teacher took a lot of time organizing the children into groups to participate in the “CLAIM Game”. When asked about this during the interview after the lesson, the teacher said:

“Organising children and preparing them for the game is difficult in a class like mine.”

An important negative finding observed during the lessons was that the use of the teachers’ guide differed from teacher to teacher in the pilot schools. One of the teachers was observed not using the guide during the lesson. The teacher only glanced through it when needed and mostly used the children’s book as the class read aloud. During our interview after the lesson, the teacher said:

“Having to use two books in a lesson is very hard. You have to look at the guide and also see what the children are reading. Why don’t we make this into one book?”

The language used in this version of the resources was still found to be a bit complicated for the children, as this was observed during the lessons when children were required to read aloud and struggled with some of the words they found difficult to read and to pronounce; for example, “surrogate outcome”. During our consultations at meetings with members of the teachers’ network, one said:

“Some of the chapters have very many new words; for example, chapter 2. Words like ‘health research’, ‘claim’, ‘experience’, ‘reason’, ‘eh’, those are so many for the children.”

During one of the pilot lessons, we observed that the teacher was hurriedly marking a few of the children’s books as they were attempting the exercises during the lesson. This allowed no time for the children to read and understand, if they were to be among the few whose books would be marked. The teacher said during the interview:

“There are so many exercises for the children to do after each lesson, it makes it very difficult for me to mark all of them.”

We observed a negative finding when one of the pilot teachers struggled to organize a class of over 100 children to participate in the CLAIM game. The children struggled to understand the instructions as they were laid out. In the interview after the lesson, the teacher said:

“I am worried about what you thought when you were watching me. I tried to organize myself last night for this activity but it was very difficult. I had to mark the children’s exercise books and also prepare for the activity. It is very difficult to do in this big class.”

### Version 2 - Positive findings

A positive finding was that the exercises in the children’s book were considered appropriate and engaging for year-5 children. One of the pilot teachers said:

“The exercises are very well suited for these children.”

Having new terms in three different languages in the textbook (English, Luganda, and Kiswahili) and explaining difficult words in a language that year-5 children could understand improved comprehension of the content being taught. A pilot teacher said:

“Using Luganda to explain the word ‘effect’ - ‘ekivaamu’ - made it simple for me to understand and then also explain to the learners.”
